# The effects of adjuvant endocrine therapy on long-term outcomes from ductal carcinoma in situ: a systematic review and meta-analysis

**DOI:** 10.1016/j.breast.2025.104521

**Published:** 2025-06-18

**Authors:** Qian Chen, Ian Campbell, Mark Elwood, Alana Cavadino, Phyu Sin Aye, Sandar Tin Tin

**Affiliations:** a Department of Epidemiology and Biostatistics, Faculty of Medical and Health Sciences, University of Auckland, Auckland, New Zealand; b Department of Surgery, Faculty of Medical and Health Sciences, University of Auckland, Auckland, New Zealand; c Department of Pharmacology, Faculty of Medical and Health Sciences, University of Auckland, Auckland, New Zealand; d Cancer Epidemiology Unit, Oxford Population Health, University of Oxford, Oxford, United Kingdom

**Keywords:** Ductal carcinoma in situ, Adjuvant endocrine therapy, Tamoxifen, Recurrence, Survival

## Abstract

**Background:**

Although adjuvant endocrine therapy (ET) is a standard treatment for hormone receptor positive ductal carcinoma in situ (DCIS), its use is variably recommended by clinicians. This paper reviewed the effects of ET in relation to recurrence and survival across diverse populations.

**Methods:**

PubMed, Embase, Web of Science, and Cochrane were searched for studies that reported outcomes of DCIS treated with ET versus no ET.

**Results:**

Three randomised trials and 42 cohort studies were included. In the trials, tamoxifen significantly reduced the risk of in-breast recurrence with a pooled hazard ratio (HR) of 0.69 (95 % CI: 0.60, 0.80). In the cohort studies, ET was associated with lower risks of any recurrence (HR 0.67; 95 % CI: 0.55, 0.83), ipsilateral breast tumour recurrence (HR 0.59; 0.51, 0.69), loco-regional recurrence (HR 0.74; 0.53, 1.02) and contralateral breast cancer (HR 0.70; 0.49, 1.00), and improved overall survival (HR 0.85; 0.79, 0.90). No significant association between ET and breast cancer specific survival was observed in either trials or cohort studies. Subgroup analyses within the cohort studies showed no significant differences in the pooled HRs for recurrence and survival by follow-up length, confounding adjustment or treatment type.

**Conclusions:**

The use of adjuvant ET reduces the risk of recurrence in patients with DCIS in clinical trials, as well as in the real-world setting. Survival benefits, however, warrant further study.

## Highlights

• Three randomised trials and 42 cohort studies were included.
• The use of adjuvant endocrine therapy (ET) reduces the risk of recurrence in patients with ductal carcinoma in situ (DCIS).
• Further research is needed to optimize patient selection for adjuvant ET.

## 1 Introduction

Ductal carcinoma in situ (DCIS) is characterised by the abnormal growth of epithelial cells within the ductal structures of the breast [1]. The standard treatment for DCIS involves mastectomy or breast-conserving surgery (BCS), with or without radiation therapy (RT) and endocrine therapy (ET), to reduce the risk of recurrence.

Approximately 65–80 % of DCIS cases are oestrogen receptor (ER) and/or progesterone receptor (PR) positive [2,3]. The international guidelines recommend testing ER status in newly diagnosed DCIS and giving endocrine therapy (ET) for ER-positive DCIS [4,5]. Yet, the use of ET, such as tamoxifen (TAM) and aromatase inhibitors, varied widely in clinical practice, ranging from 11 % to 71 % [3,[6], [7], [8], [9], [10]]. This suggests that clinicians are yet to be convinced by the efficacy of treatment, and/or women and clinicians feel the side effects of treatment outweigh the benefits. Previous meta-analyses assessed the efficacy and safety outcomes of postoperative TAM by pooling results from only two randomised controlled trials (RCTs) that enrolled patients prior to 2000 [11,12]. Importantly, there is a lack of evidence on the efficacy of ET in relation to recurrence and survival across diverse populations in the real-world setting.

We therefore conducted a systematic review and meta-analysis of published RCTs and observational cohort studies to assess the long-term outcomes of adjuvant ET use in women with DCIS, as well as to examine whether these outcomes differ across subgroups.

## 2 Methods

This review was conducted in accordance with the Preferred Reporting Items for Systematic Reviews and Meta-Analysis (PRISMA) statement [13].

### 2.1 Search strategy and eligibility criteria

A systematic literature search was undertaken in Medline, Embase, Web of Science, and Cochrane library up until the February 18th, 2025, using the following keywords and phrases: DCIS, ductal carcinoma in situ, breast event, recurrence, endocrine therapy, tamoxifen, anastrozole, aromatase inhibitor, in combinations of ‘AND’ or ‘OR’ (see details in the Supplementary Table 1).

The pre-defined eligibility criteria for the systematic review were: (1) RCTs or observational cohort studies, (2) involved women diagnosed with DCIS, with no evidence of invasion or nodal involvement, (3) reported information on the use of adjuvant ET as the exposure of interest (with no dose or duration requirement), and (5) published in English as a full text article. We excluded systematic reviews, case reports and conference abstracts. For multiple publications that reported the same outcome(s) from the same study, we included the most recent or comprehensive results.

The meta-analysis included studies that reported hazard ratio (HR) and corresponding 95 % confidence interval or p-value [14] for the outcomes of interest, or the number of events that allowed for the approximation of HR using the method described by Watkins and Bennett [15].

### 2.2 Data extraction and quality assessment

The data were extracted by QC and studies with unclear eligibility were further reviewed by STT. Any discrepancies were resolved through discussion. The following information was extracted: study first author, number of patients included, year published, country, data accrual period, length of follow-up, study type, treatment types, the proportion of hormone receptor positive patients, adjusted confounders, and outcomes. When studies reported both multivariable and univariable HR, the multivariable HR was used; otherwise, the univariable HR was used.

The risk of bias was assessed by the Newcastle-Ottawa Scale (NOS) for non-randomised studies [16]. The NOS comprises eight items assessing three aspects: selection of study population, comparability of groups, and outcome for cohort studies. The NOS has a maximum score of 9, with scores higher than 7 indicating good quality, and 5–7 indicating moderate quality [16].

### 2.3 Study outcomes

The primary outcome was in-breast recurrence, which referred to DCIS recurrence and/or invasive cancer in either the ipsilateral and/or contralateral breast as reported in the RCTs. As the cohort studies did not report data on in-breast recurrence, any recurrence was assessed as the outcome, which was defined as the recurrence of DCIS and/or invasive cancer in either breast, lymph nodes, or distant metastasis; studies that reported disease free survival (DFS) were also included in this analysis.

The secondary outcomes included ipsilateral breast tumour recurrence (IBTR), loco-regional recurrence (LRR), contralateral breast cancer (CBC), breast cancer specific survival (BCSS), and overall survival (OS). IBTR was defined as subsequent development of DCIS recurrence and/or invasive cancer in the ipsilateral breast. DCIS-IBTR and invasive-IBTR were analysed separately for studies that reported relevant results. LRR was defined as recurrence of DCIS and/or invasive cancer in the ipsilateral breast and/or regional lymph nodes. CBC was defined as subsequent development of DCIS and/or invasive cancer in the contralateral breast.

### 2.4 Statistical analysis

Random-effects meta-analyses were undertaken, and the pooled HR and 95 % CI were weighted by generic inverse variance. For analyses involving more than 10 studies, Egger's test and funnel plots were used to assess the likelihood of publication bias. If the corresponding p-value from Egger's test was less than 0.05, a leave-one-out sensitivity analysis was conducted to examine whether any individual study had excessive influence on the pooled estimate.

For cohort studies, subgroup analyses were undertaken by hormone receptor status (ER and/or PR-positive, any status), length of follow-up (<5 years, ≥5 years), adjustment for confounding (univariable, multivariable) and treatment type (BCS alone, BCS with RT) for the outcomes with at least two studies in each group. All analyses were conducted in R version 4.4.2 [17]. Statistical tests were two-sided, using a significance threshold of p-value less than 0.05.

## 3 Results

### 3.1 Study selection

**Fig. 1:**
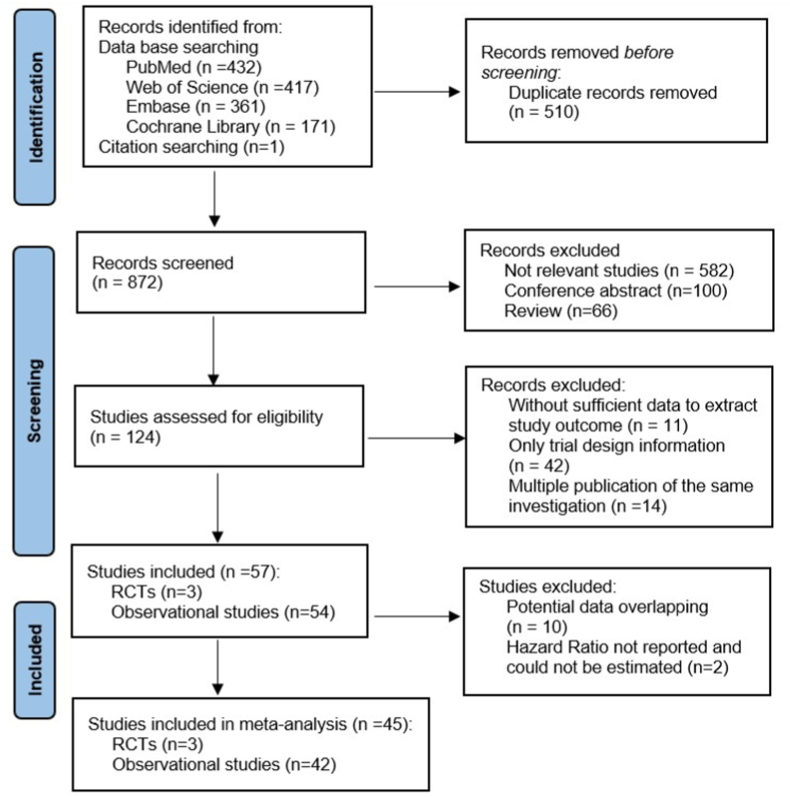
Flow diagram for study selection according to PRISMA.

A total of 1382 records were identified (Fig. 1). After exclusion of duplicates and non-relevant records, 57 studies (3 RCTs and 54 cohort studies) were included in the systematic review. The results from 45 studies (3 RCTs and 42 cohort studies) were meta-analysed for the outcomes of interest.

### 3.2 Study characteristics

Three RCTs compared the use of adjuvant tamoxifen in women with DCIS treated with BCS, with or without RT (Table 1). The UK/ANZ [18] and NSABP B-24 [19] trials investigated standard-dose tamoxifen for 5 years in women with DCIS of any ER status. In contrast, the TAM-01trial [20] examined low-dose (5 mg per day) tamoxifen for 3 years in women with intraepithelial neoplasia (69 % DCIS). Notably, TAM-01 trial only included women with ER-positive DCIS and those with unknown ER status.

**Table 1:** Characteristics of RCTs.

Study (Country)	Enrolment period	Sample size	Patient characteristics	Median follow-up (years)	Treatment groups	Outcomes included for meta-analysis
UK/ANZ [18] (UK, Australia, NZ)	1990–1998	1701	any ER status	12.7	BCS ± RT + TAM (20 mg/day for 5 years) vs. BCS ± RT	DCIS-IBTR, invasive IBTR; in-breast event
NSABP B24 [19] (UK)	1991–1994	1804	any ER status	13.6	BCS + RT + TAM (20 mg/day for 5 years) vs. BCS + RT	DCIS-IBTR, invasive IBTR; in-breast event
TAM-01 (US) [20]	2008–2015	346	ER positive or unknown	9.7	BCS ± RT + TAM (5 mg/day for 3 years) vs. BCS ± RT	in-breast event

Abbreviations: BCS: breast conserving surgery; ER: oestrogen receptor; IBTR: ipsilateral breast tumour recurrence; NSABP: National Surgical Adjuvant Breast and Bowel Project; NZ: New Zealand; RT: radiation therapy; TAM: Tamoxifen. UK: United Kingdom; US: United States.

Of the 42 [[8], [21], [22], [23], [24], [25], [26], [27], [28], [29], [30], [31], [32], [33], [34], [35], [36], [37], [38], [39], [40], [41], [42], [43], [44], [45], [46], [47], [48], [49], [50], [51], [52], [53], [54], [55], [56], [57], [58], [59], [60], [61]] cohort studies included in the meta-analysis, 39 [[8], [22], [23], [24], [25], [26], [27], [28], [29], [30], [31], [34], [35], [36], [37], [38], [39], [40], [41], [42], [43], [44], [45], [46], [47], [48], [49], [50], [51], [52], [53], [54], [55], [56], [57], [58], [59], [60], [61]] used a retrospective design and 3 [21,32,33] used a prospective design (secondary analysis of the trial data) Table 2. Ten studies [8,23,29,31,35,36,40,43,44,50] were population-based, 11 [[21], [25], [26], [27], [32], [33], [37], [38], [46], [56], [60]] were multi-centre studies, and the remaining were conducted in a single centre. Six [23,27,31,43,45,50] studies reported outcomes for hormone receptor positive DCIS, and the remaining studies reported outcomes for DCIS with any receptor status. The NOS scores were high, ranging from 7 to 9 (Supplementary Table 2).

**Table 2:** Characteristics of observational cohort studies included in the systematic review.

Study, published year (Country)	Data source^a^	Data period	Sample size	Hormone receptor and other key feature	Median or mean follow-up^b^ (years)	Treatment groups^c^	Adjusted covariates^d^	Outcomes included for meta-analysis^e^
Wright et al.,2024 (US) [21]	E5194 trial	1997–2002	665	NA	19.2	BCS ± RT + TAM vs. BCS ± RT	Cohort, lesion size	IBTR
Wang et al. 2024 (China) [22]	SYSUCC	2010–2017	483	71.4 % ER/PR+	8.4	Surgery ± RT + ET vs. Surgery ± RT	Univariate	LRR, OS all recurrence
Poli et al., 2024 (US) [23]	NCDB	2004–2017	69961	HR+, ≥50 years, low/intermediate grade	6	Surgery + ET vs. Surgery	age, race, insurance status, facility type, comorbidity score, tumour size	OS
Choi et al., 2024 (Korea) [24]	Seoul National University Hospital	2003–2016	544	NA	7.8	BCS ± RT + ET vs. BCS ± RT	age, size, density, margin, RT, morphology, ultrasound pattern	IBTR
Vicini et al. 2023 (US, AU, Sweden) [25]	UUH, UMASS, KPNW, RMH	1996–2011	923	NA	8.8	BCS ± RT + ET vs. BCS ± RT	age, grade, size, biosignature risk groups, RT	LRR
Schmitz et al., 2023 (UK, US) [26]	Sloane, MDACC, NCDB	1999–2017	32638	NA	6.7	BCS ± RT + ET vs. BCS ± RT	age, year of diagnosis, cohort	DCIS-IBTR, iIBTR
Niu et al. 2023 (China) [27]	Eight hospitals	2006–2016	935	HR+	7.2	Mastectomy + ET vs. Mastectomy	Univariate	All recurrence
Dreyfuss et al. 2023 (US) [28]	MSKC	2004–2018	1675	NA	4.2	BCS + RT + ET vs. BCS + RT	age, RT boost, tumour grade	LRR
Sousa et al. 2023 (Brazil) [29]	FOSP	2000–2016	2192	NA	4.1	BCS + RT + ET vs. BCS + RT	age and education	All recurrence, OS, BCSS
Yang et al., 2022 (China) [30]	Fudan Cancer Centre	2008–2016	291	83.1 % ER/PR+	5.5	BCS ± RT + ET vs. BCS ± RT	Univariate	IBTR, CBC, all recurrence
Oses et al. 2023 (Spain) [62]	Hospital Clinic Barcelona	1999–2019	207	74.4 % ER+	10.6	BCS ± RT + ET vs. BCS ± RT	age, family history, presentation, RT, nuclear grade, comedonecrosis, margin	Excluded as it reported IBTR as OR (0.4, 95 %CI 0.9–1.4)
Tsai et al., 2022 (Taiwan) [31]	Taiwan Cancer Registry	2010–2017	1611	ER+, 96.1 % low/intermediate grade	3.3	BCS + RT + ET vs. BCS + RT	residential areas, diagnosed year, insurance income rank	All recurrence
Chua et al., 2022 (11 developed countries) [32]	BIG 3–07/TROG 07.01 trial	2007–2014	1608	Non-low-risk DCIS	6.6	BCS ± RT + ET vs. BCS ± RT	age, RT, menopausal status, presentation, tumour size, grade, margin	IBTR
McCormick et al., 2021 (US) [33]	RTOG 9804 trial	1999–2016	629	Low-risk DCIS	13.9	BCS ± RT + TAM vs. BCS ± RT	Treatment arm, age, margins, tumour size, nuclear grade	IBTR
Livingston-Rosanoff et al., 2021 (US) [34]	Wisconsin Cancer Centre	1997–2006	536	54 % ER+	10	BCS ± RT + ET vs. BCS ± RT	Univariate	LRR
Hwang et al., 2021 (Korea) [35]	KBCR	2000–2014	11404	79 % HR+	5	Surgery+/RT + TAM vs. Surgery ± RT	ER, PR, age, BMI, surgery period, surgery, RT	OS
Byun et al., 2021 (US) [36]	NCDB	2004–2016	24424	≤50 years, 72.6 % ER+	5.4	BCS ± RT + ET vs. BCS ± RT	Treatment groups, race, Charlson–Deyo, grade, ER status, margins, tumour size, income, education, year of diagnosis, insurance	OS overall; OS in ER + subgroup
Lewis et al., 2021 (US) [63]	NCDB	2004–2015	1927	HER2+, 63 % HR+	3.0	Surgery ± RT ± HER2-therapy + ET vs. Surgery ± RT ± HER2-therapy	HER2-targeted therapy, age, Charlson-Deyo Score, ER status	Excluded due to potential overlap with Poli et al., 2024 in reported OS
Ahn et al., 2021 (Korea) [64]	KBCR	1997–2019	2307	HER2+	6	Surgery ± RT + ET vs. Surgery ± RT	age, family history, surgery, PR receptor	Excluded due to potential overlap with Hwang et al., 2021 in reported OS
Halasz et al., 2021 (US) [65]	NCDB	2004–2016	607	71.5 % ER+	NA	Surgery ± RT + ET vs. Surgery ± RT	age, race, grade, size, endocrine receptor, RT, surgery	Excluded due to potential overlap with Byun et al., 2021 in reported OS
Cambra et al., 2020 (Spain) [37]	Cancer centres in Catalonia	1993–2011	624	44.9 % ER+	8.8	BCS ± RT + TAM vs. BCS ± RT	year of diagnosis, re-excision, tumour size, margins, boos dose	IBTR
Mannu et al., 2020 (UK) [3]	NHSBSP	2000–2014	943	ER+	15	Surgery ± RT + ET vs. Surgery ± RT	year of diagnosis, age at diagnosis, time since diagnosis, tumour size, DCIS grade, laterality, treatments	Excluded as it reported BCSS as rate ratio (1.33, 95 % CI 0.72–2.49)
Lazzeroni et al., 2020 (Italy) [66]	IEO	1997–2007	649	78 % ER+	8.7	BCS+/RT + TAM vs. BCS+/RT	age, necrosis, ER, PR, Her-2, Ki-67, size, grade	Excluded due to potential overlap with Guerrieri-Gonzaga et al., 2016 in reported IBTR
Meattini et al., 2019 (Italy) [38]	Nine Italian centres	1997–2012	1072	77.8 % ER+	8.4	BCS + RT + ET vs. BCS + RT	postmenopausal, ER+, PR+, margins	IBTR
Cho et al., 2019 (Korea) [39]	Samsung medical	1996–2009	209	Tumour size ≤1 cm, 77.5 % HR+	8.7	BCS ± RT + ET vs. BCS ± RT	Tumour size, age, comedo type, nuclear grade, subtypes, margin, multifocal tumour, RT	IBTR
Thompson et al., 2018 (UK) [40]	NHSBSP	2003–2012	7007	NA	5,3	BCS ± RT + ET vs. BCS ± RT	Multivariable factors were not specified	IBTR, all recurrence
Shurell et al., 2018 (US) [41]	MSKC	1980–2010	1294	NA	6.6	BCS + RT + ET vs. BCS + RT	menopausal status, RT dose, presentation, necrosis,	IBTR
Kuo et al., 2018 (Taiwan) [42]	NTUH	2003–2010	207	81.1 % ER+	7.9	BCS + TAM vs. BCS alone	age, ER status, combined VNPI score/E5194 trial risk category	IBTR, all recurrence
Hwang et al., 2018 (Korea) [43]	KBCS	2000–2014	5919	Luminal A subtype (ER/PR+, HER2−)	4.7	Surgery ± RT + TAM vs. Surgery ± RT	ER, PR, age, operation period, operation, RT	OS in ER + subgroup
Corradini et al., 2018 (Germany) [44]	MCR	1998–2014	1048	67.7 % HR+	7.3	BCS ± RT + ET vs. BCS ± RT	age, RT, multifocality, margin	LRR, OS
Chaudhry et al., 2018 (Canada) [8]	BCOU	2009–2014	2336	66 % ER+	4.2	Surgery ± RT + ET vs. Surgery ± RT	Propensity score matching	All recurrence, OS
De Lorenzi et al., 2018 (Italy) [67]	IEO	2000–2008	419	63 % ER+	9.2	BCS + RT + TAM vs. BCS + RT	Treatments, Menopausal, Family history, BMI, histological subtype, necrosis, Microcalcifications, Multifocality, margin	Excluded due to potential overlap with Guerrieri-Gonzaga et al., 2016 in reported IBTR
Leonardi et al., 2018 (Italy) [68]	IEO	2000–2010	123	67 % ER/PR+	10.7	BCS + RT + ET vs. BCS + RT	Univariate	Excluded due to potential overlap with Guerrieri-Gonzaga et al., 2016 in reported IBTR
Chaudhary et al., 2018 (US) [45]	Wisconsin Cancer Centre	2002–2015	482	ER+	5.2	Surgery+/RT + ET vs. Surgery+/RT	ER/PR status, Surgery/Radiation	All recurrence
Moran et al., 2017 (Canada US, France) [46]	10 academic institutions	1980–2010	3704	30 % ER+	9	BCS + RT + TAM vs. BCS + RT	age, margin, grade, tumour size	IBTR
Miller et al., 2017 (US) [47]	MSKC	1978–2011	2720	NA	6.8	BCS ± RT + ET vs. BCS ± RT	age, family history, presentation, grade, surgery year, radiation	CBC
Isfahanian et al., 2017 (Canada) [48]	TOHCC	2003–2008	348	55 % ER+	5.4	BCS + RT + ET vs. BCS + RT	Univariate	IBTR
Hill et al., 2017 (US) [49]	Dartmouth Cancer Centre	2000–2010	366	55 %ER+	7	BCS ± RT + ET vs. BCS ± RT	age, MRI, size, ER-positive, RT	IBTR
Guerrieri-Gonzaga et al., 2016 (Italy) [50]	IEO	1997–2008	883	ER+	7.7	BCS ± RT + TAM vs. BCS ± RT	age, RT, BMI, margins, grade	In-breast recurrence; IBTR, DCIS-IBTR, iIBTR, CBC
Cronin et al., 2016 (US) [51]	MSKC	1978–2010	2634	NA	6.3	BCS ± RT + ET vs. BCS ± RT	age, presentation, family history, necrosis, excision number, margin, RT, surgery year	DCIS-IBTR
Subhedar et al., 2015 (US) [69]	MSKC	1978–2010	2558	NA	6.3	BCS ± RT + ET vs. BCS ± RT	age, family history, presentation, RT, nuclear grade, margin, treatment period	Excluded due to potential overlap with Dreyfuss et al., 2023 in reported all recurrence
Van Zee et al., 2015 (US) [70]	MSKC	1978–2010	2698	NA	6.3	BCS ± RT + ET vs. BCS ± RT	presentation, family history, necrosis, number of excisions, margin status, RT, treatment period	Excluded due to potential overlap with Dreyfuss et al., 2023 in reported all recurrence
Lo et al., 2015 (Canada) [52]	University of British Columbia	2000–2009	2061	28.6 % ER+	8	BCS + RT + ET vs. BCS + RT	margin, size, age, grade, ER status	LRR, all recurrence, OS
Wang et al., 2014 (Singapore) [53]	Singapore General Hospital	1992–2011	716	51.3 % ER+	5.9	BCS ± RT + ET vs. BCS ± RT	Univariate	IBTR
Sweldens et al., 2014 (Belgium) [54]	University Hospital Leuven	1973–2010	467	NA	7.2	BCS ± RT + ET vs. BCS ± RT	age, margin	IBTR
Pilewskie et al., 2014 (US) [71]	MSKC	1997–2010	2212	NA	4.9	BCS ± RT + ET vs. BCS ± RT	age, menopausal status, family history, presentation, margin, excision number, RT	Excluded due to potential overlap with Dreyfuss et al., 2023 in reported LRR
Lee et al., 2013 (US) [55]	St Luke's-Roosevelt Hospital Centre	1990–2009	294	52.4 % ER+	5.3	BCS ± RT + TAM vs. BCS ± RT	age, tumour size, grade, margin, VNPI score, RT	IBTR
Hathout et al., 2013 (Canada) [56]	HMR, MUHC, CHUQ	2003–2010	380	71 % ER+	4.4	BCS + RT + ET vs. BCS + RT	Univariate	IBTR
Bailes et al., 2013 (US) [57]	MDACC	1996–2009	1903	46 % ER+	4.8	Surgery ± RT + ET vs. Surgery ± RT	age, race, menopausal Status, HRT, family history, Presenting, size, grade, ER status, surgery, RT	All recurrence
Yi et al., 2012 (US) [58]	MDACC	1990–2007	794	NA	7.1	BCS ± RT + ET vs. BCS ± RT	age at diagnosis, family history, presentation, RT, nuclear grade, necrosis, margins, excision number, year of surgery	IBTR
Alvarado et al., 2012 (US) [59]	MDACC	1996–2006	2037	NA	5.2	BCS ± RT + TAM vs. BCS ± RT	age, presentation, ER status, margin, grade, RT	LRR
Rudloff et al., 2010 (US) [72]	MSKC	1991–2006	1842	NA	5.6	BCS ± RT + ET vs. BCS ± RT	age, family history, presentation, grade, surgery year, RT	Excluded due to potential overlap with Shurell et al., 2018 in reported IBTR
Habel et al., 2009 (US) [60]	KPNC, KPSC, HPHC	1990–2001	1407	NA	4.8	BCS + TAM vs. BCS	Diagnosis year	IBTR, all recurrence
Yau et al., 2006 (Hongkong) [61]	PYNEH	1994–2003	75	NA	5.1	BCS + RT + ET vs. BCS + RT	Univariate	IBTR

Abbreviations: AU: Australia; BCS: breast conserving surgery; BCOU: British Columbia Breast Cancer Outcomes Unit; BMI: body mass index; CBC: contralateral breast cancer; CHUQ: Centre Hospitalier Universitaire de Québec; CN: China; ER: oestrogen receptor; ET: oestrogen therapy; FOSP: Fundação Oncocentro de São Paulo; HER2: human epidermal growth factor receptor 2; IBTR: ipsilateral breast tumour recurrence; IEO: European Institute of Oncology; KBCS: Korean Breast Cancer Society; KPNC: Kaiser Permanente of Northern California; KPSC: Kaiser Permanente of Southern California; KPNW: Kaiser Permanente Northwest; NTUH: National Taiwan University Hospital; MDACC: MD Anderson Cancer Centre; MSKC: Memorial Sloan Kettering Cancer Centre; MUHC: McGill University Health Centre; MUR: Munich Cancer Registry; NA: not available; NCDB: National Cancer Database; NHSBSP: NHS Breast Screening Programme; OS: overall survival; PR: progesterone receptor; RMH: Royal Melbourne Hospital and Royal Women's Hospital; RT: radiation therapy; SYSUCC: Sun Yat-sen University Cancer Centre; TAM: Tamoxifen; TOHCC: The Ottawa Hospital Cancer Centre; UK: United Kingdom; UUH: Uppsala University Hospital; UMASS: University of Massachusetts, Worcester; US: United States; VNPI: Van Nuys Prognostic Index; PYNEH: Pamela Youde Nethersole Eastern Hospital; HMR: Hôpital Maisonneuve-Rosemont; HPHC: Harvard Pilgrim Health Care; LRR: locoregional recurrence.

e. Outcomes included for meta-analysis: If the study did not include into the meta-analysis, the reasons were stated.

a Data source: for prospective design trial, the trial code specified, otherwise, the agency of registry or the research centre was recorded.

b Follow-up: If the study reported both median and mean follow-up, the median follow-up was recorded.

c Treatment groups: If the study specified the type of surgery or endocrine therapy, the specific type was recorded; otherwise, it was recorded as surgery or endocrine therapy.

d Adjusted covariates: the covariates were specified for multivariate adjusted outcomes, otherwise, it recorded as univariate.

e Outcomes included for meta-analysis: If the study did not include into the meta-analysis, the reasons were stated.

### 3.3 Meta-analysis of RCTs

**Fig. 2:**
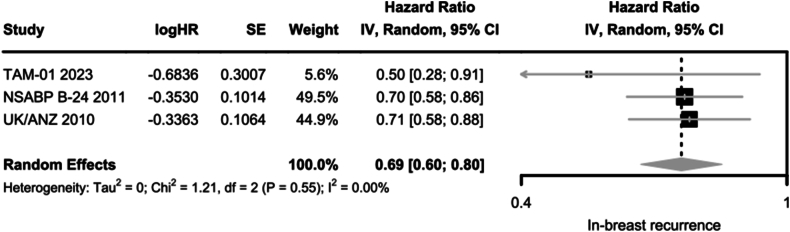
Risk of any in-breast recurrence in patients who received Tamoxifen in comparison to those who did not.

In the meta-analysis of three RCTs [[18], [19], [20]], the adjuvant TAM reduced the risk of any in-breast recurrence compared to no TAM (HR 0.69; 95 %CI, 0.60 to 0.80) (Fig. 2). The lowest HR was seen in the trial with predominantly ER-positive DCIS [20]. Two RCTs also reported the results for IBTR and CBC separately; the pooled results showed a statistically significant reduction in the risk of DCIS-IBTR and CBC among patients who received TAM, but the effect was not significant for invasive IBTR (Supplementary Fig. 1).

The NSABP B-24 [19] trial was the only RCT that reported locoregional/distant recurrence, BCSS, and OS, and found no significant difference (locoregional/distant recurrence, HR 0.66, 95 % CI: 0.25, 1.73; BCSS, HR 0.81, 95 %CI: 0.43, 1.50; OS, HR 0.86, 95 %CI: 0.66, 1.11). The UK/ANZ [18] trial reported that the use of TAM had no significant effect on BCSS or OS.

### 3.4 Meta-analysis of observational cohort studies

#### 3.4.1 In-breast recurrence

Only one study assessed in-breast recurrence and reported a significant association of low-dose TAM with a reduced risk in ER-positive DCIS (HR 0.70; 95 % CI:0.54, 0.91) [50].

#### 3.4.2 Any recurrence

**Fig. 3:**
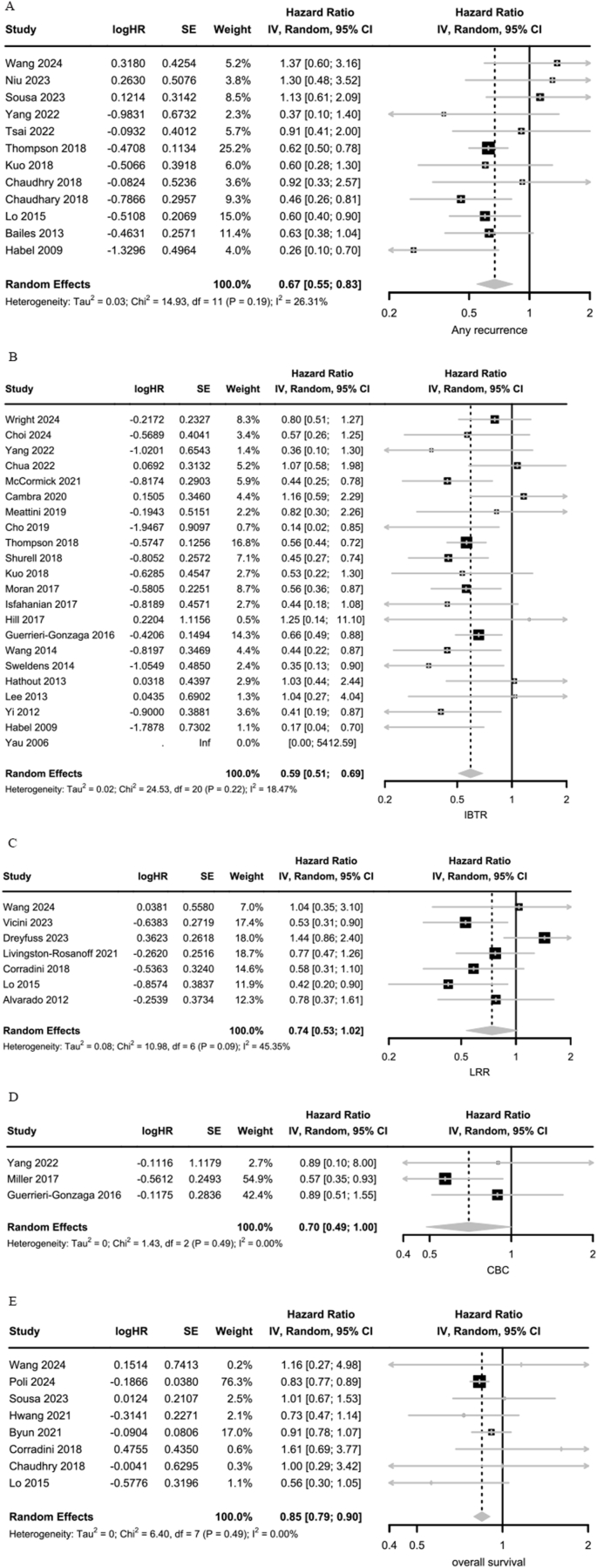
Association between the endocrine therapy and any recurrence (A), ipsilateral breast tumour recurrence (B), loco-regional recurrence (C), contralateral breast cancer (D), and overall survival (E).

The pooled results of 12 studies showed that ET was significantly associated with a lower risk of any recurrence (HR 0.67; 95 % CI: 0.55, 0.83) (Fig. 3A).

#### 3.4.3 Ipsilateral breast tumour recurrence (IBTR)

In the meta-analysis of 22 studies, ET was significantly associated with a lower IBTR (HR: 0.59; 95 % CI: 0.51, 0.69) (Fig. 3B).

Four studies reported DCIS-IBTR, and three reported invasive IBTR. ET was associated with a lower risk of DCIS-IBTR and invasive IBTR although the difference for the latter was not statistically significant (Supplementary Fig. 2). Additionally, a population-based study in UK found a stronger association in patients with ER-positive DCIS, with a rate ratio of 0.62 (95 % CI: 0.49, 0.80) [3].

#### 3.4.4 Loco-regional recurrence (LRR)

The pooled results of seven studies showed that adjuvant ET was associated with a marginal decreased risk of LRR (HR 0.74; 95 % CI: 0.53, 1.02) (Fig. 3C).

#### 3.4.5 Contralateral breast cancer (CBC)

In the meta-analysis of three studies, patients treated with adjuvant ET was associated with a borderline reduced risk of CBC compared to those who were not (HR 0.70; 95 % CI: 0.49,1.00) (Fig. 3D).

#### 3.4.6 Breast cancer specific survival (BCSS)

A Brazilian study (median follow-up of 4.1 years) showed no significant difference in BCSS in patients who received adjuvant ET compared to those who did not (HR 0.78, 95 % CI: 0.29–2.12); ER status was not known [29]. Similarly, a UK study (median follow-up of 15 years) reported no BCSS benefit from ET versus no ET in screen-detected ER-positive DCIS, with a rate ratio of 1.33 (95 % CI: 0.72, 2.49) [3].

#### 3.4.7 Overall survival (OS)

When the results from eight studies were pooled, with median follow-up ranging from 4.1 to 9.0 years, ET was significantly associated with improved OS (HR 0.85, 95 % CI: 0.79, 0.90) (Fig. 3E).

#### 3.4.8 Subgroup analysis

There were no significant differences in the pooled HR of any recurrence and OS by HR status, length of follow-up, adjustment for confounding, or treatment type (Table 3, Supplementary Table 3).

**Table 3:** Subgroups analysis of association of endocrine therapy with any recurrence and overall survival, by hormone receptor status or length of follow-up.

Variable	Any recurrence				OS			
	No. of studies	HR (95 %CI)	I^2^ (%)	P value^a^	No. of studies^b^	HR (95 %CI)	I^2^ (%)	P value^a^
**Hormone receptor status**								
Any status	9	0.67 (0.53,0.83)	26.67	0.751	5	0.93 (0.67,1.28)	6.33	0.669
ER/PR+	3	0.74 (0.40,1.39)	49.99		3	0.85 (0.70,1.04)	65.71	
**Median follow-up years**								
<5 years	5	0.72 (0.47,1.12)	42.36	0.611	3	0.80 (0.50, 1.26)	38.24	0.676
5–10 years	7	0.64 (0.51,0.80)	17.58		5	0.89 (0.74,1.07)	44.22	

ER: oestrogen receptor; HR: hazard ratio; OS: overall survival; PR: progesterone receptor.

a P-value for subgroup differences.

#### 3.4.9 Publication bias and sensitivity analysis

Egger's test and funnel plots showed no publication bias for IBTR and any recurrence. In sensitivity analyses, removing one study at a time from the pooled analysis did not substantially affect the results (Supplementary Figs. 3–6). Moreover, the pooled HR for OS did not change substantially after excluding Poli et al. [23], which is the largest study included in this analysis (Supplementary Fig. 7).

## 4 Discussion

In this meta-analysis involving patients with DCIS, use of adjuvant ET significantly reduced the risk of in-breast recurrence in the RCTs and was also associated with lower risks of any recurrence, IBTR, LRR, and CBC in the cohort studies. Although we found better OS in the cohort studies, the results from RCTs do not support this. There was also no significant association between ET and BCSS in either RCTs or cohort studies.

Our updated meta-analysis of the three RCTs demonstrated that TAM significantly reduced in-breast recurrence by 30 %, an outcome that has not been previously reported. Notably, the UK/ANZ [18] and NSABP B-24 [19] trials were conducted before the routine evaluation of hormone receptor status in DCIS, whereas the TAM-01 trial [20] only included ER-positive (or unknown status) DCIS. In a subgroup analysis of the NSABP B-24 trial [73], which focused on ER-positive DCIS, showed a 42 % reduction in the in-breast recurrence, comparable to the 50 % reduction reported in the TAM-01 trial [20]. The non-significant invasive IBTR reduction in the TAM group may be attributed to the substantial reduction in IBTR that has been achieved through RT [74], and the lack of targeting ER-positive DCIS in the 2 larger RCT [18,19].

Consistent with the results from RCTs, our meta-analysis of cohort studies showed that ET was associated with a lower risk of any recurrence, LRR and CBC in women with DCIS by approximately 30 %. Moreover, ET resulted in a 40 % lower risk of IBTR, with statistically significant associations observed in the pooled analysis of DCIS-IBTR. Of note, the Memorial Sloan Kettering Cancer Centre DCIS nomogram included adjuvant ET, together with other clinicopathologic risk factors in the prognostic nomogram for predicting IBTR risk in patients with DCIS treated with BCS [72]. A US population-based study found that the 10-year in-breast recurrence rate in women with DCIS was lower in those treated with ET, with rates of approximately 16 %, 10 % and 8 % for those treated with BCS + RT, BCS + ET, and BCS + RT + ET, respectively [75].

Our review of cohort studies did not find improved BCSS associated with ET as reported in the RCTs [18,19]. While we found a significant OS benefit associated with ET, this may just reflect that ET is more likely to be utilised by patients in overall good health [76]. Given the high BCSS in women with DCIS (e.g., >96 % in low-risk DCIS) [77,78], demonstrating additional survival benefits from ET remains challenging.

ER/PR-positive and human epidermal growth factor receptor 2 (HER2)-negative DCIS has a more favourable prognosis compared to other molecular subtypes [79]. The subgroup analysis of the NSABP B-24 trial showed that TAM significantly improved breast cancer free survival only in patients with ER-positive DCIS [73]. Similar findings were reported in the pooled analysis by the Early Breast Cancer Trialists' Collaborative Group (EBCTCG) [80]. Our subgroup analysis of cohort studies, however, showed no significant differences based on hormone receptor status, possibly because we were only able to compare ER-positive DCIS and DCIS with any ER status.

Age and/or menopausal status, and tumour grade at DCIS diagnosis have been shown to be important prognostic factors [26,72]. Although we were unable to perform subgroup analysis based on these factors, most cohort studies included in our meta-analysis accounted for these variables in the multivariable models. Unlike postoperative RT following BCS, which consistently has been shown to reduce IBTR regardless of these factors [26,74], some studies have reported that ET may offer greater benefit in specific populations. The UK/ANZ [18] trial showed that TAM was more effective in reducing in-breast event among women aged over 60 or those with low-intermediate grade DCIS compared to those aged under 60 or those with high grade, although this difference may be due to differences in ER/PR status which was not available in this study. Similarly, Guerrieri-Gonzaga et al. found that the association of low-dose TAM with lower IBTR in ER-positive DCIS was significantly greater in women aged over 50 compared to those under 50 [50]. The TAM-01 [20] trial showed that low-dose TAM appears to be more effective in postmenopausal women compared to premenopausal women, although the difference was not statistically significant. This contrasts with the effect of standard dose adjuvant TAM for invasive cancer [80].

Women who were treated with adjuvant ET alone were more likely to have low-risk (low and/or intermediate grade, small tumour size) ER-positive DCIS, and therefore were expected to have lower recurrence rates [75]. In the Comparing an Operation to Monitoring, With or Without Endocrine Therapy for Low-Risk DCIS (COMET) trial involving women with low-risk DCIS, about 98–99 % were ER positive, and 71 % received ET in the active monitoring group and 66 % in the guideline-concordant care group; the study reported non-inferior risk of invasive IBTR at 2 years in the active monitoring group but was not designed to assess the effects of ET [10]. Another ongoing RCT [81] is evaluating whether the post-BCS low-dose TAM is comparable to RT in preventing IBTR in low-risk ER-positive DCIS.

Similar to the UK/ANZ [18] trial, our meta-analysis of cohort studies found no significant difference in the associations of ET with recurrence between patients who underwent BCS alone and those who underwent BCS with RT.

In the pooled analysis by EBCTCG, the effect of TAM was more pronounced in the trials with a 5-year treatment duration and longer follow-up [80] However, our observational findings did not differ by length of follow-up. While we were unable to undertake subgroup analysis by ET treatment duration or adherence, one study reported that using ET for at least three years was significantly associated with a lower risk of LRR, compared to no ET or use for less than three years [34]. Another study from the US also showed lower recurrence rates associated with good adherence to ET in women aged over 65 years with DCIS, who in general had favourable prognosis [82]. Non-adherence to ET has also been associated with worse survival outcomes in patients with early breast cancer [83].

Types of ET may influence outcomes from DCIS. While we were not able to examine the effects of different types of ET (e.g., aromatase inhibitors vs. TAM) on outcomes from DCIS in the real-world setting, results from the RCTs indicate that anastrozole may be superior to TAM in postmenopausal women younger than 60 years (NSABP B-35 trial) [84], or at least non-inferior (International Breast Cancer Intervention Study-II (IBIS-II) trial [85]).

The benefits of ET as breast cancer chemoprevention in healthy women at high-risk have been established in the RCTs [[86], [87], [88], [89]]. While there has been no trial conducted in patients with DCIS, our meta-analysis, showing a borderline reduced risk of CBC and significant decreased risk of any breast event after adjuvant ET, supports this.

This is the first meta-analysis that included results from real-world evidence on the association of ET use with recurrence and survival outcomes in women with DCIS. Our analysis, however, was restricted to studies published in English, predominantly from Europe and North America, which may limit the generalizability of the findings.

In summary, the use of adjuvant ET reduced the risk of recurrence in patients with DCIS. However, further research is needed to identify specific DCIS subgroups who might benefit from adjuvant ET in terms of survival outcomes.

## CRediT authorship contribution statement

**Qian Chen:** Writing – review & editing, Writing – original draft, Methodology, Formal analysis, Data curation, Conceptualization. **Ian Campbell:** Writing – review & editing, Methodology. **Mark Elwood:** Writing – review & editing, Methodology. **Alana Cavadino:** Writing – review & editing, Methodology. **Phyu Sin Aye:** Writing – review & editing, Methodology. **Sandar Tin Tin:** Writing – review & editing, Methodology, Funding acquisition, Conceptualization.

## Ethics approval and consent to participate

No ethical approval and patient consent are required, since all analyses were based on previously published studies.

## Data availability

Data used in this review is provided in the manuscript and Supplementary appendices.

## Funding information

This study was supported by 10.13039/501100001511Auckland Medical Research Foundation (Ref: 1124002) and University of Auckland Research Development Fund (Ref: 3729227). The funding sources had no direct involvement in this study or the decision to submit the paper for publication.

## Declaration of competing interest

The authors declare no competing interests.
