# Supplementary material for: The effects of adjuvant endocrine therapy on long-term outcomes from ductal carcinoma in situ: a systematic review and meta-analysis
Source: Breast. 2025 Jun 18;82:104521. doi: 10.1016/j.breast.2025.104521 (PMC12221685; doi:10.1016/j.breast.2025.104521)
Supplement: Multimedia component 1 [file mmc1.docx]

**Supplemental Materials**

Supplementary Table 1. Searching strategies

| **Database** | **Search String** | **Results** |
| --- | --- | --- |
| MEDLINE (PubMed) | (((((DCIS[Title/Abstract]) OR (ductal carcinoma in situ[Title/Abstract])) AND ((((((ipsilateral[Title/Abstract]) OR (contralateral[Title/Abstract])) OR (breast cancer[Title/Abstract])) OR (breast event[Title/Abstract])) OR (recurrence[Title/Abstract])) OR (relapse[Title/Abstract]))) AND ((((endocrine) OR (tamoxifen)) OR (anastrozole)) OR (aromatase inhibitor))) AND (English[Language])) NOT (((Case reports[Publication Type]) OR (Review[Publication Type])) OR (Comment[Publication Type])) | 432 |
| Embase | ((DCIS or ductal carcinoma in situ).ti. and (recurrence or breast event or relapse or survival).ab. and (endocrine or tamoxifen or anastrozole or aromatase inhibitor).af. and English.lg.) not (Letter or Books or Preprint or Review).pt. | 417 |
| Web of Science | ((((TI=(DCIS or ductal carcinoma in situ)) AND AB=(recurrence or breast event or breast cancer)) AND ALL=(endocrine or tamoxifen or anastrozole or aromatase inhibitor)) AND LA=(English)) NOT DT=(Letter OR Review OR Withdrawn Publication) | 361 |
| Cochrane library | (DCIS or ductal carcinoma in situ):ti,ab,kw AND (recurrence or breast event or relapse or event or survival):ti,ab,kw AND (endocrine or tamoxifen or aromatase inhibitor or anastrozole) AND English:la NOT (case reports or comment or letters or review):pt | 171 |
| Citation searching |  | 1 |

Supplementary Table 2. Newcastle-Ottawa Scale (NOS) of observational cohort studies included for the meta-analysis

| **Study** | **Representativeness** | **Selection** | | | **Comparability** | **Outcome** | | | **Total score** |
| --- | --- | --- | --- | --- | --- | --- | --- | --- | --- |
|  |  | **Selection** | **Ascertainment of Exposure** | **Outcome** |  | **Assessment of Outcome** | **Follow-Up** | **Adequacy** |  |
| Poli 2024 | 1 | 1 | 1 | 1 | 2 | 1 | 1 | 1 | 9 |
| Wright 2024 | 1 | 1 | 1 | 1 | 2 | 1 | 1 | 1 | 9 |
| Wang 2024 | 1 | 1 | 1 | 1 | 0 | 1 | 1 | 1 | 7 |
| Choi 2024 | 1 | 1 | 1 | 1 | 2 | 1 | 1 | 1 | 9 |
| Dreyfuss 2023 | 1 | 1 | 1 | 1 | 2 | 1 | 1 | 1 | 9 |
| Schmitz 2023 | 1 | 1 | 1 | 1 | 2 | 1 | 1 | 1 | 9 |
| Niu 2023 | 1 | 1 | 1 | 1 | 0 | 1 | 1 | 1 | 7 |
| Sousa 2023 | 1 | 1 | 1 | 1 | 2 | 1 | 1 | 1 | 9 |
| Vicini 2023 | 1 | 1 | 1 | 1 | 2 | 1 | 1 | 1 | 9 |
| Yang 2022 | 1 | 1 | 1 | 1 | 0 | 1 | 1 | 1 | 7 |
| Chua 2022 | 1 | 1 | 1 | 1 | 2 | 1 | 1 | 1 | 9 |
| Tsai 2022 | 1 | 1 | 1 | 1 | 2 | 1 | 1 | 1 | 9 |
| McCormick 2021 | 1 | 1 | 1 | 1 | 2 | 1 | 1 | 1 | 9 |
| Byun 2021 | 1 | 1 | 1 | 1 | 2 | 1 | 1 | 1 | 9 |
| Hwang 2021 | 1 | 1 | 1 | 1 | 2 | 1 | 1 | 1 | 9 |
| Livingston-Rosanoff 2021 | 1 | 1 | 1 | 1 | 0 | 1 | 1 | 1 | 7 |
| Cambra 2020 | 1 | 1 | 1 | 1 | 2 | 1 | 1 | 1 | 9 |
| Meattini 2019 | 1 | 1 | 1 | 1 | 2 | 1 | 1 | 1 | 9 |
| Cho 2019 | 1 | 1 | 1 | 1 | 2 | 1 | 1 | 1 | 9 |
| Hwang 2018 | 1 | 1 | 1 | 1 | 2 | 1 | 1 | 1 | 9 |
| Thompson 2018 | 1 | 1 | 1 | 1 | 2 | 1 | 1 | 1 | 9 |
| Shurell 2018 | 1 | 1 | 1 | 1 | 2 | 1 | 1 | 1 | 9 |
| Kuo 2019 | 1 | 1 | 1 | 1 | 2 | 1 | 1 | 1 | 9 |
| Corradini 2018 | 1 | 1 | 1 | 1 | 2 | 1 | 1 | 1 | 9 |
| Chaudhry 2018 | 1 | 1 | 1 | 1 | 2 | 1 | 1 | 1 | 9 |
| Chaudhary 2018 | 1 | 1 | 1 | 1 | 2 | 1 | 1 | 1 | 9 |
| Moran 2017 | 1 | 1 | 1 | 1 | 2 | 1 | 1 | 1 | 9 |
| Miller 2017 | 1 | 1 | 1 | 1 | 2 | 1 | 1 | 1 | 9 |
| Isfahanian 2017 | 1 | 1 | 1 | 1 | 0 | 1 | 1 | 1 | 7 |
| Hill 2017 | 1 | 1 | 1 | 1 | 2 | 1 | 1 | 1 | 9 |
| Guerrieri-Gonzaga 2016 | 1 | 1 | 1 | 1 | 2 | 1 | 1 | 1 | 9 |
| Cronin 2016 | 1 | 1 | 1 | 1 | 2 | 1 | 1 | 1 | 9 |
| Lo 2015 | 1 | 1 | 1 | 1 | 2 | 1 | 1 | 1 | 9 |
| Wang 2014 | 1 | 1 | 1 | 1 | 0 | 1 | 1 | 1 | 7 |
| Sweldens 2014 | 1 | 1 | 1 | 1 | 0 | 1 | 1 | 1 | 7 |
| Bailes 2013 | 1 | 1 | 1 | 1 | 2 | 1 | 1 | 1 | 9 |
| Hathout 2013 | 1 | 1 | 1 | 1 | 0 | 1 | 1 | 1 | 7 |
| Lee 2013 | 1 | 1 | 1 | 1 | 2 | 1 | 1 | 1 | 9 |
| Yi 2012 | 1 | 1 | 1 | 1 | 2 | 1 | 1 | 1 | 9 |
| Alvarado 2012 | 1 | 1 | 1 | 1 | 2 | 1 | 1 | 1 | 9 |
| Habel 2009 | 1 | 1 | 1 | 1 | 1 | 1 | 1 | 1 | 8 |
| Yau 2006 | 1 | 1 | 1 | 1 | 0 | 1 | 1 | 1 | 7 |

| A | 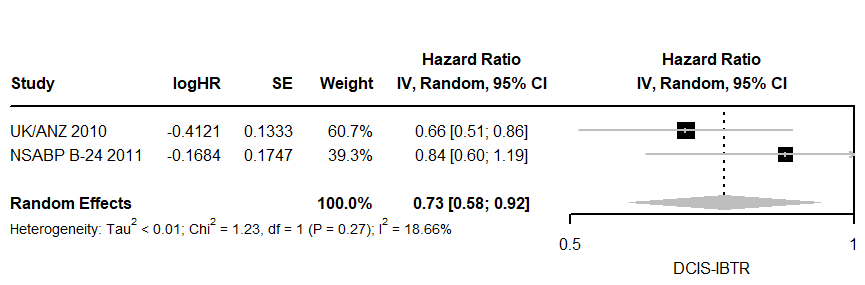 |
| --- | --- |
| B | 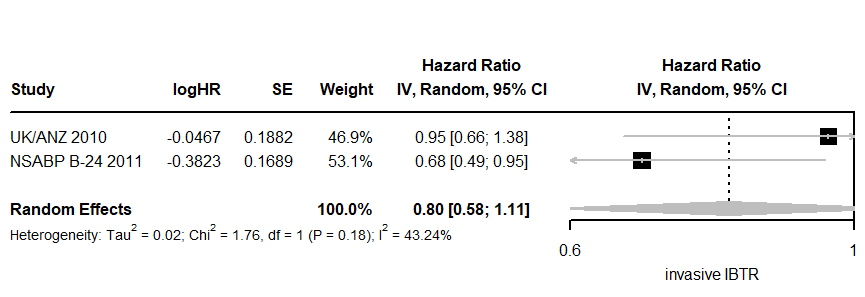 |
| C | 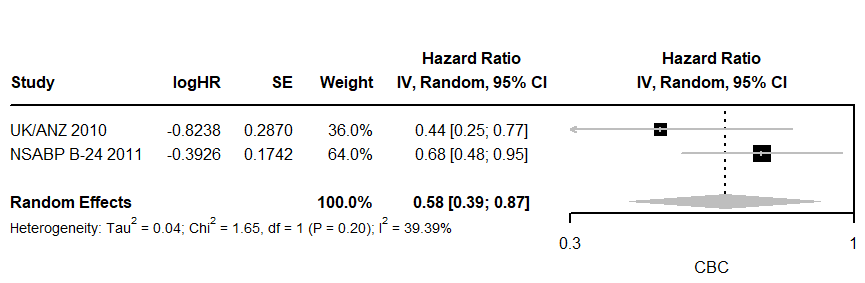 |

Supplementary Figure 1. Risk of DCIS-ipsilateral breast tumour recurrence (A), invasive ipsilateral breast tumour recurrence (B), contralateral breast cancer (C) in patients who received endocrine therapy in comparison to those who did not.

| A | 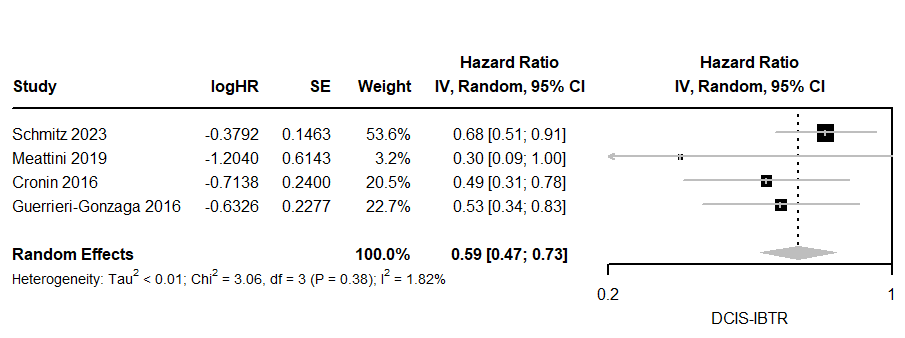 |
| --- | --- |
| B | 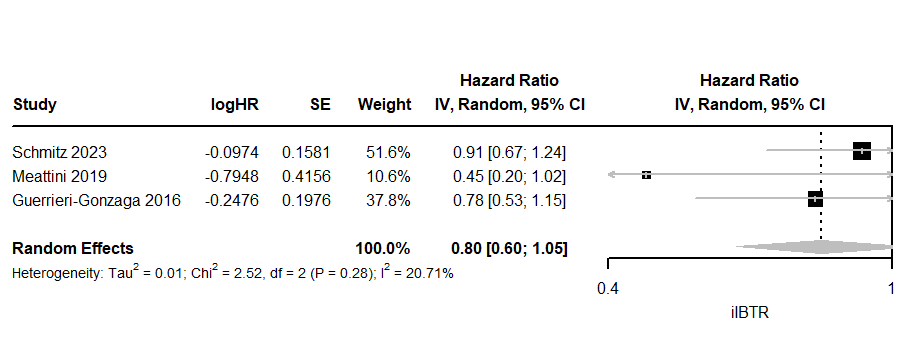 |

Supplementary Figure 2. Association between the endocrine therapy and DCIS-ipsilateral breast tumour recurrence (A) and invasive ipsilateral breast tumour recurrence (B).

Supplementary Table 3. Subgroups analysis of association between the endocrine therapy and ipsilateral breast tumour recurrence, any recurrence and loco- regional recurrence, by adjustment for confounding, and treatment types.

| **Variable** | **IBTR** | | | | **Any recurrence** | | | | **LRR** | | | |
| --- | --- | --- | --- | --- | --- | --- | --- | --- | --- | --- | --- | --- |
|  | **No. of studies** | **HR (95%CI)** | **I^2^**  **(%)** | **P value^a^** | **No. of studies** | **HR (95%CI)** | **I^2^**  **(%)** | **P value^a^** | **No. of studies** | **HR (95%CI)** | **I^2^**  **(%)** | **P value^a^** |
| **Study variables** | | | | | | | | |  |  |  |  |
| Univariable | 5 | 0.53  (0.34,0.83) | 2.9 | 0.62 | 3 | 1.00  (0.49, 2.04) | 31.7 | 0.22 | 2 | 0.80 (0.51,1.27) | 0.0 | 0.65 |
| Multivariable | 16 | 0.60  (0.50,0.72) | 29.1 |  | 9 | 0.63  (0.53,0.76) | 12.6 |  | 5 | 0.70 (0.44,1.10) | 62.3 |  |
| **Treatment types** | | | | | | | | | | | | |
| BCS alone | 2 | 0.56 (0.42,0.74) | 44.9 | 0.40 | 2 | 0.87 (0.62, 1.21) | 36.0 | 0.17 | NA |  |  |  |
| BCS+RT | 5 | 0.34 (0.11,0.42) | 0.0 |  | 3 | 0.42 (0.19, 0.94) | 41.0 |  | NA |  |  |  |

BCS: breast conserving surgery; HR: hazard ratio; IBTR: ipsilateral breast tumour recurrence; LRR: locoregional recurrence; NA: not applicable; RT: radiation therapy.

1. P-value for subgroup differences.


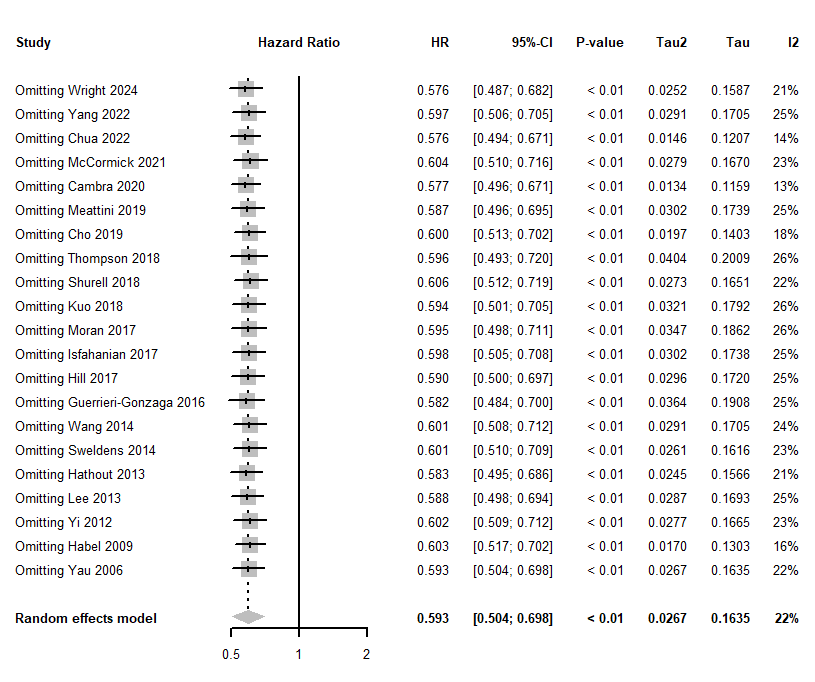


Supplementary Figure 3. Leave-one-out sensitivity analysis of IBTR.


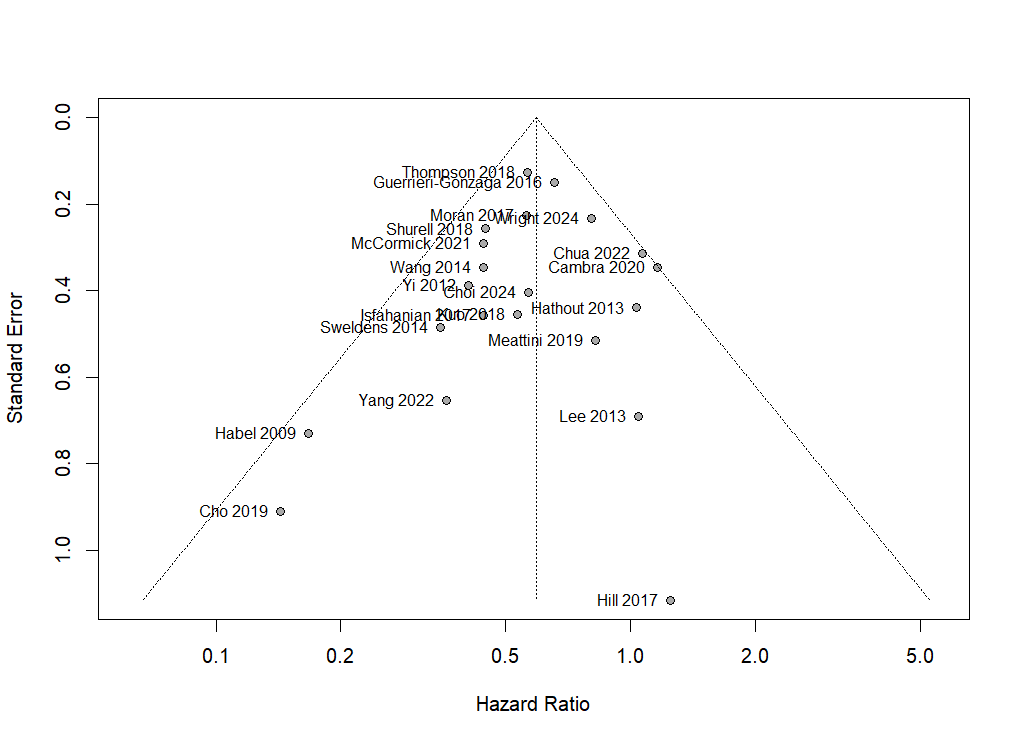


Note: Yau et al. 2006 was excluded due to values deemed too small by the linear regression test

Supplementary Figure 4. Funnel plot of standard error by hazard ratio of IBTR, t value of Egger’s test =-0.68, P = 0.507.


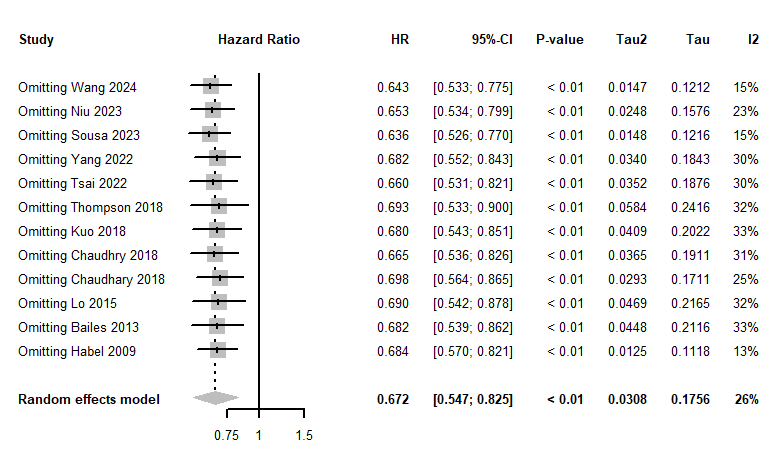


Supplementary Figure 5. Leave-one-out sensitivity analysis of any recurrence.


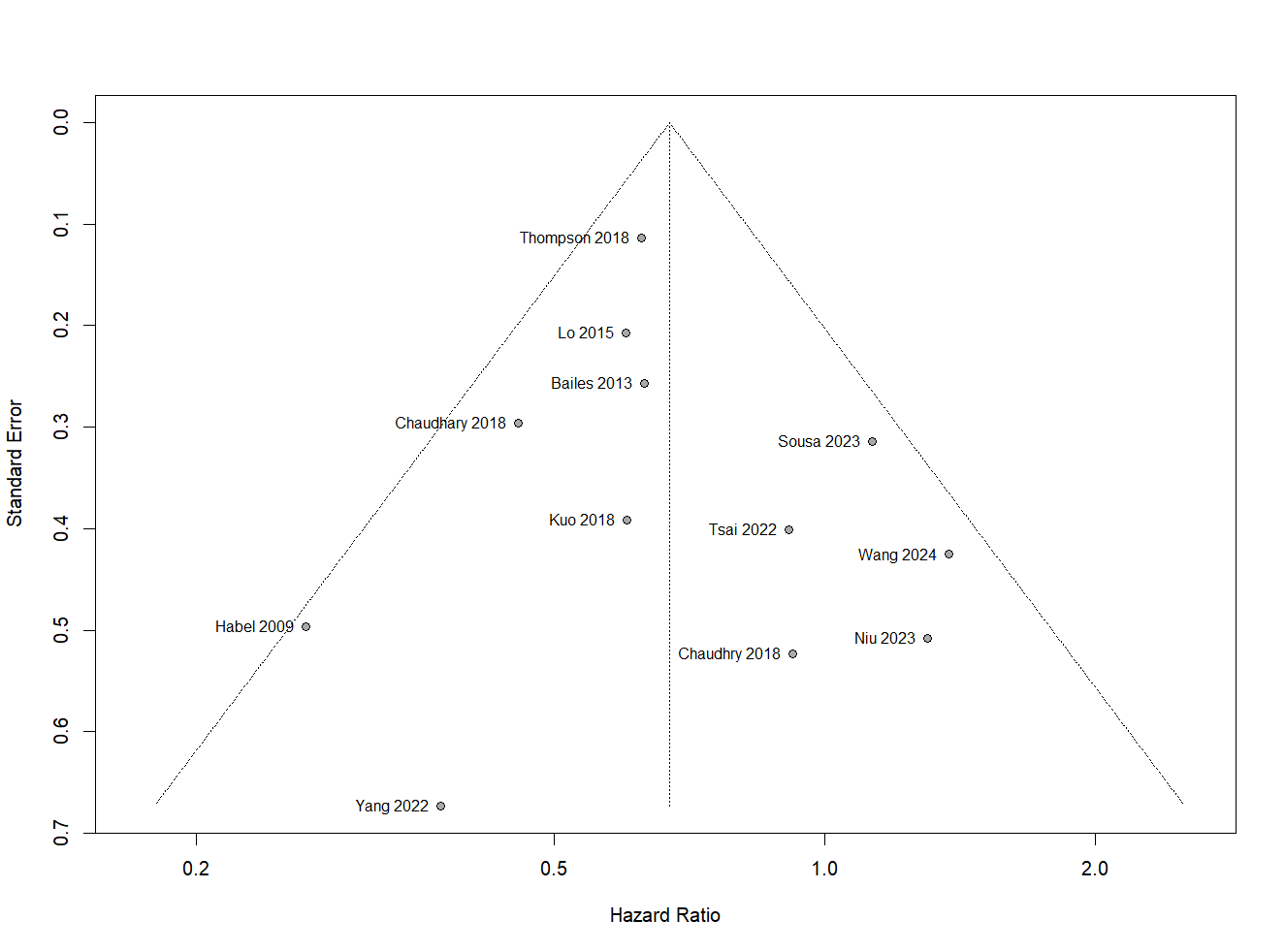


Supplementary Figure 6. Funnel plot of standard error by hazard ratio of any recurrence, t value of Egger’s test =0.61, P = 0.554.


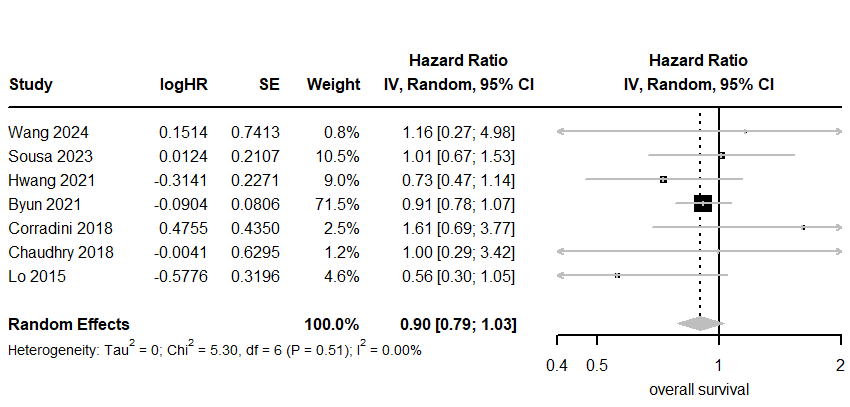


Supplementary Figure 7. Association between the endocrine therapy and overall survival, after excluding Poli et al. 2024
